# 100 Long Term Outcomes for Inpatient Visits in Bcqp - Feasibility to Bridge the Gap

**DOI:** 10.1093/jbcr/irad045.073

**Published:** 2023-05-15

**Authors:** Joan Weber, Claudia Malic, QBR Outpatient Work Group, Brian Lynch

**Affiliations:** ABA, Tewksbury, Massachusetts; Childrens’ Hospital of Eastern Ontario, Ottawa, Ontario; ABA, Dallas, Texas; BData Inc., Minneapolis, Minnesota; ABA, Tewksbury, Massachusetts; Childrens’ Hospital of Eastern Ontario, Ottawa, Ontario; ABA, Dallas, Texas; BData Inc., Minneapolis, Minnesota; ABA, Tewksbury, Massachusetts; Childrens’ Hospital of Eastern Ontario, Ottawa, Ontario; ABA, Dallas, Texas; BData Inc., Minneapolis, Minnesota; ABA, Tewksbury, Massachusetts; Childrens’ Hospital of Eastern Ontario, Ottawa, Ontario; ABA, Dallas, Texas; BData Inc., Minneapolis, Minnesota

## Abstract

**Introduction:**

The ABA burn registry is designed to improve the quality and cost of burn care by collecting and exchanging information on burn injuries and outcomes. The BCQP has data related to inpatients visits only. Data on long term outcomes from outpatient settings are not yet available in the ABA’s Quality Burn Registry, but there is agreement that such data should be collected. With any expansion of the registry, attention should be given to the type of data that are collected and the efforts needed to gather accurate data. A pilot phase (alpha pilot) is required before embarking on a larger scale of data gathering.

**Aim:**

This alpha pilot aims to determine what outcomes should be selected to continue data collection for patients who were discharged from a burn center and are followed up within outpatient settings.

**Methods:**

The QBR Outpatient Work Group identified 9 items (5 clinical and 4 psychosocial outcomes) that could define the long term outcomes after discharge from the initial admission related to the thermal injuries. A five questions survey was developed to evaluate the length of time spent by registrars to gather the data, and around data quality assurance. Seven verified burn centres were enrolled on voluntary basis on the alpha pilot. Each centre had to enter a patient in each of the predefined categories (age and burn extent related) for the 12 months post discharge divided in 3 monthly periods. The data gathering finished in April 2022.

**Results:**

A total of 29 patients were entered in the alpha pilot, with 5 patients < 18 yrs of age and 6 over the age 65. The most frequent cause of burn injury was flame (18/29). Fourteen patients sustained burn injuries >20% TBSA. The 95% wound closure was documented in 79.3% and follow up was complete or ongoing for 89.7%. Details about any surgical procedures after discharge was well documented (100%). The burn scar appearance was present in the chart in 69.2% of the cases and only in 3 patients pressure garments was not documented. Presence of depression or PTSD was not documented in 50% of the cases, whereas the presence of pain and itch was documented between 79% to 83%. Registrars found that it took up to 20 minutes to retrieve the data from the charts.

**Conclusions:**

The findings were presented to the Quality and Burn Registry Committee. As alpha pilot was the first PDSA cycle for this project, further amendments were made to the definitions and layout of the data. A beta pilot (14 hospitals) is ongoing currently as second PDSA cycle in order to identify further barriers for large scale implementation of long term outcomes in the burn registry.

**Applicability of Research to Practice:**

implementation of long term outcomes in the ABA Burn Registry

